# High throughput detection of capillary stalling events with Bessel beam two-photon microscopy

**DOI:** 10.1117/1.NPh.10.3.035009

**Published:** 2023-09-12

**Authors:** John Giblin, Sreekanth Kura, Juan Luis Ugarte Nunuez, Juncheng Zhang, Gulce Kureli, John Jiang, David A. Boas, Ichun A. Chen

**Affiliations:** a Boston University, Department of Biomedical Engineering, Boston, Massachusetts, United States; b Boston University, Neurophotonics Center, Boston, Massachusetts, United States

**Keywords:** Bessel beam, capillary stalling, cerebral blood flow, two-photon microscopy

## Abstract

**Significance:**

Brief disruptions in capillary flow, commonly referred to as capillary “stalling,” have gained interest recently for their potential role in disrupting cerebral blood flow and oxygen delivery. Approaches to studying this phenomenon have been hindered by limited volumetric imaging rates and cumbersome manual analysis. The ability to precisely and efficiently quantify the dynamics of these events will be key in understanding their potential role in stroke and neurodegenerative diseases, such as Alzheimer’s disease.

**Aim:**

Our study aimed to demonstrate that the fast volumetric imaging rates offered by Bessel beam two-photon microscopy combined with improved data analysis throughput allows for faster and more precise measurement of capillary stall dynamics.

**Results:**

We found that while our analysis approach was unable to achieve full automation, we were able to cut analysis time in half while also finding stalling events that were missed in traditional blind manual analysis. The resulting data showed that our Bessel beam system was captured more stalling events compared to optical coherence tomography, particularly shorter stalling events. We then compare differences in stall dynamics between a young and old group of mice as well as a demonstrate changes in stalling before and after photothrombotic model of stroke. Finally, we also demonstrate the ability to monitor arteriole dynamics alongside stall dynamics.

**Conclusions:**

Bessel beam two-photon microscopy combined with high throughput analysis is a powerful tool for studying capillary stalling due to its ability to monitor hundreds of capillaries simultaneously at high frame rates.

## 1 Introduction

Adequate blood flow is essential to supporting healthy brain function.^1^ Global and regional reductions^2^^–^^6^ in cerebral blood flow as well as alterations in hemodynamic^3^^,^^6^ responses are associated with or precede cognitive decline across multiple neurodegenerative diseases, including Alzheimer’s and Parkinson’s diseases. At the smallest scale, alterations in microvascular structure^7^ and flow can also drive pathology,^8^^–^^10^ including regions affected by stroke induced hypoxia. This includes transient disruptions in flow, or “stalls,” in normally continuous cerebral capillary flow.^11^^–^^13^ Recent work has demonstrated the potential role of stalls in pathology, including Alzheimer’s disease^14^ and stroke.^15^^–^^18^ In addition to potentially inducing local hypoxia, these events can contribute to total vascular resistance,^14^ as well as flow heterogeneity,^19^ further inducing hypoxia.^20^ Treatments to reduce this stalling have been associated with favorable cognitive outcomes in a model of Alzheimer’s^14^ and reduced infarct size in stroke.^15^^,^^18^

Investigations into the effects of stalling and its potential as a therapeutic target have been hindered due to technological limitations. Traditional two-photon imaging z-stacks are frequently used to measure stalling rates^12^^,^^14^^–^^17^ but only observe individual capillaries for a small fraction of the acquisition time. Further, even when a stall is observed, the onset and cessation of events is typically not seen. The alternative is to monitor a single XY plane over a long duration, but due to two-photon confinement, only a handful of capillaries are typically visible in a single plane. Optical coherence tomography (OCT) angiography provides continuous volumetric monitoring of capillaries^13^^,^^18^ but the OCT systems used to study stalling have lacked the temporal resolution to catch shorter stalling events [though faster OCT angiography (OCTA) rates have been demonstrated^21^ but not yet applied to measuring stalls]. In addition, OCT also lacks the ability to perform multicolor fluorescence imaging like two-photon microscopy, which has aided in investigating the source of the stalling events.^14^^,^^15^^,^^17^^,^^18^

In addition to these limitations, analyzing and extracting stalling information for these enormous datasets is highly labor intensive. For both two-photon microscopy and OCT, images must be manually analyzed to identify the stalling events. In the case of two-photon microscopy, analysis must be crowd sourced to citizen scientists who volunteer to sift through hundreds of thousands of capillary images to identify stalled capillaries so that the resulting statistics can be reported in a reasonable amount of time.^14^^,^^22^ Further, the sparse nature of stalling in these data makes the construction of a large training dataset for automation difficult due to class imbalance. As more complex investigations are performed and therapeutic modulation of stalling continues to be explored, the amount of data that needs analysis will grow significantly. Therefore, there is a need for high throughput imaging and analysis of stalling parameters to avoid a data processing bottleneck.

To address these challenges, we use a custom Bessel beam two-photon microscope^23^ to identify stalling events by continuously monitoring a 713×713×120  μm volume acquired at 0.57 Hz, roughly four times the volumetric imaging rate of conventional OCT angiograms. We then developed a correlation-based detection approach to semi-automate the analysis for stall identification, which reduced the time required to analyze the data by 50% and led to a 2 to 3 times improvement in the number of stalls identified when compared with unassisted analysis. With the combined improvements in temporal resolution and improved detection rate, we found that a majority of stalls are very short in duration, making it likely they were missed in other studies. This enabled us to observe differences in stall dynamics between young and aged mice as well as track changes in stall dynamics before and after stroke in the same capillary network.

## 2 Results

**Fig. 1:**
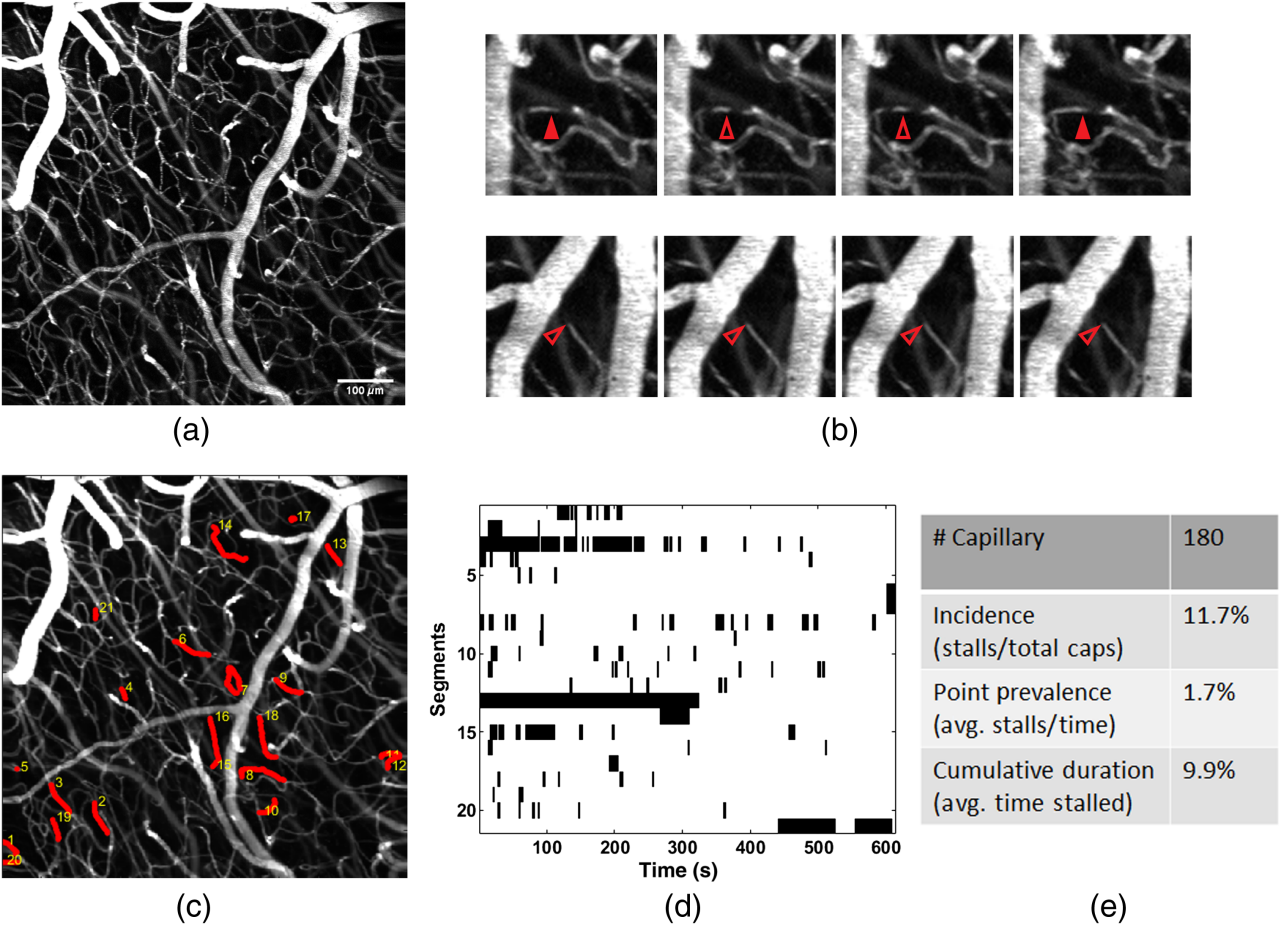
(a) Maximum intensity projection (MIP) of a 713×713×120  μm volume scanned at 0.57 Hz for 10 min to observe instances of capillary stalling. (b) Representative examples of types at stalling events captured during imaging. (Top) RBC shadows are stationary for two sequential frames during a short stalling event before normal flow resumes. (Bottom) A shadow is continuously present in the same position along the length of the capillary during a long stalling event. (c) MIP with stalled capillaries marked in red. (d) Stallogram shows the times each capillary was stalled (marked in black). (e) Stall statistics calculated based on the stallogram and total number of capillaries (Video 1, mp4, 7.54 MB [URL: https://doi.org/10.1117/1.NPh.10.3.035009.s1]).

We used a home built, axicon-based, Bessel beam two-photon microscope to image cortical vasculature over an extended depth of field^24^ [Fig. 1(a)]. Within this volume, roughly 200 capillaries are monitored for both short- and long-duration stalling events [Fig. 1(b) and Video 1], where red blood cell (RBC) shadows are stationary for at least two or more consecutive frames. Identification of stalled capillaries from 10-min time courses, and the times they were stalled are used to calculate key stall parameters^13^ [Fig. 1(c)]. This includes the percent of all capillaries that undergo stalling at any time (incidence), the average percent of capillaries actively stalling at a given time (point prevalence), and the average total time a stalled capillary spends stalling during the measurement duration (cumulative stall duration). Identification of stalling capillaries and annotating the time and duration of each stall event for the acquired time series data is a particularly laborious task since there are hundreds of capillaries to observe at higher volume rates than previous studies. To make stalls more apparent, the data is broken down into sub-ROIs containing ∼20 to 30 capillaries each, which is then scrolled through frame by frame to identify instances where all RBC shadows within a capillary are stationary between two or more frames. This process is repeated for all sub-ROIs until the whole field of view has been processed. A full 713×713×120  μm dataset of 350 frames took close to 12 h per dataset and the results can be variable depending on the focus and fatigue level of the observer. To improve both throughput and detection accuracy, we introduced a metric that can predict the occurrence of a stall in each capillary.

**Fig. 2:**
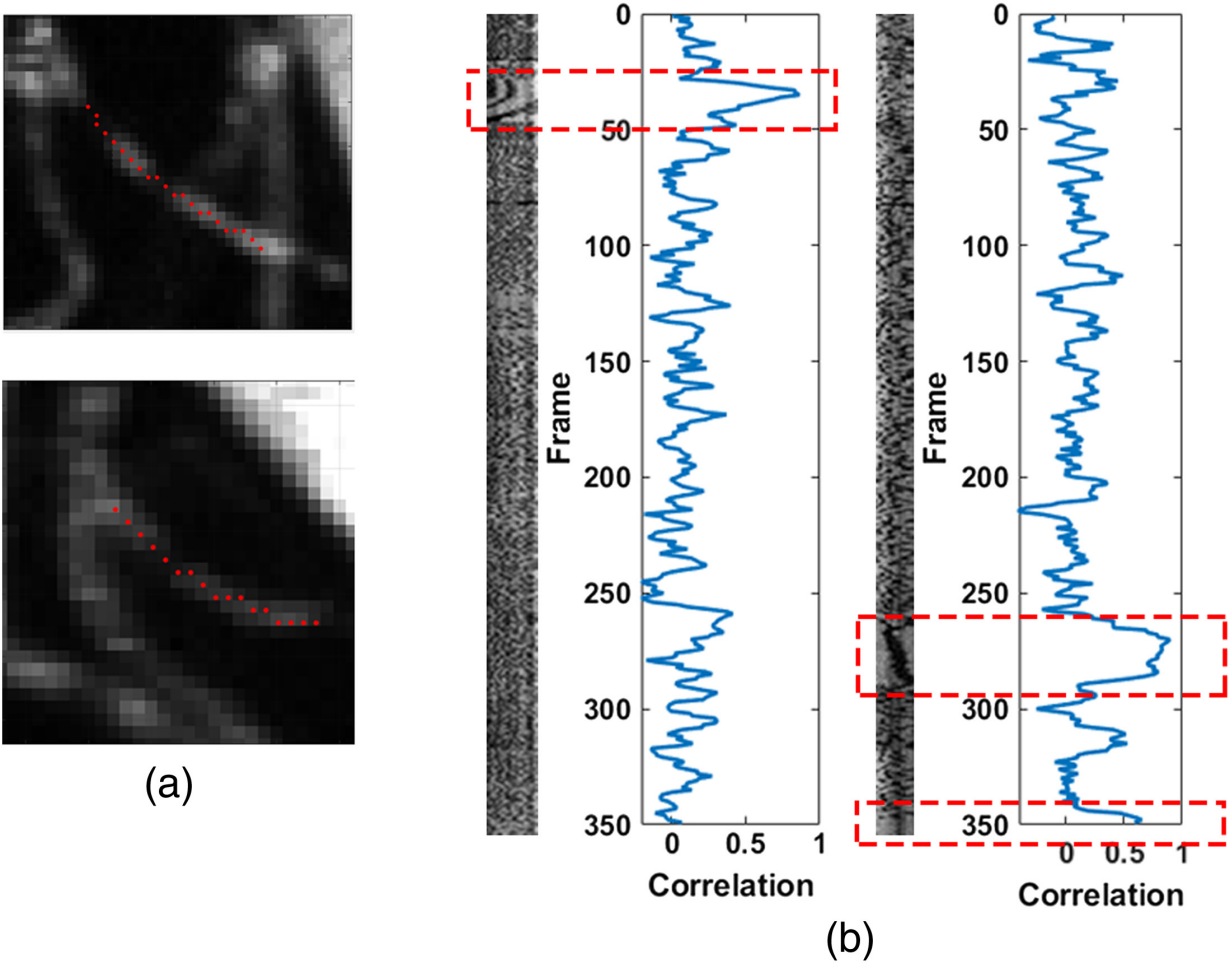
(a) Capillaries that undergo stalling with the centerline drawn automatically after single click selection. (b) LT plots of the capillaries from (a) with the correlation versus time plotted alongside. Red boxes indicate times where the capillary was stalled and the same RBCs are present for multiple frames, which corresponds to a rise in frame-to-frame correlation.

During normal flow, at the scanning rate used, new shadows (mostly RBCs) will be present in every frame, varying in number and position. During a stall, the same shadows will remain in the same position along the length of the capillary until flow resumes in that capillary. Therefore, the intensity along the length of the vessel is expected to be well correlated during stalling events. To facilitate extraction of the intensity along the length of the capillary, we semi-automatically extract the centerline trace of capillaries of interest^25^ [Fig. 2(a)]. This generates images of intensity along the capillary length versus time (LT images). Plotting the frame-to-frame intensity correlation versus time shows a clear increase during stalling events, before returning to lower levels [Fig. 2(b)]. To validate this metric, we threshold the correlation and use it as a predictor of stalling, which was then confirmed by a human observer.

### 2.1 Correlation Validation

To validate our approach, we first manually analyzed 10 datasets from 4 mice (4- to 8-months old). For each dataset, we then used our correlation-based approach to predict stalls from LT images generated for each capillary. The threshold was set intentionally low, trading-off specificity for increased sensitivity. The resulting prediction was then analyzed by another observer to determine if flagged events by either the manual inspection or correlation prediction were a stall (true positive) or not (false positive) (Table 1). This thresholded correlation generated significantly more false positives than true positives, meaning that it could not be directly used for calculation of stall statistics. However, while this correlation-based identification approach was unable to reliably predict stall parameters accurately, we found that the validation process was roughly twice as fast as the original blind manual analysis (dropping from 10 to 12 h to ∼6  h per ROI for an experienced observer). It could therefore be used to improve analysis throughput. In addition, we found 2 to 3 times as many stalling events (true positives) with this approach indicating that the manual process results in a large number of missed stalling events (false negatives). Further, the few false negatives for the correlation-based approach (stalls found manually but not flagged by correlation) give confidence that we are capturing as many stalling events as possible and that we do not need to confirm capillaries not flagged by the correlation-based approach, further speeding the manual validation process. This correlation-based identification approach can therefore be used as an analysis aid that can speed up the analysis process while providing more precise estimation of the stalling statistics.

**Table 1:** Truth tables of capillaries that stall found via manual inspection of the data (top) and correlation thresholding of the LT plot (bottom). Ground truth determination in all cases was determined by a separate expert observer wherever the manual and/or correlation-based approaches marked a stall.

	Manual negative	Manual positive
Ground truth negative (2111)	98.2% (2074)	1.8% (37)
Ground truth positive (347)	70.6% (245)	29.4% (102)
	Correlation negative	Correlation positive
Ground truth negative (2111)	45.5% (961)	55.5% (1150)
Ground truth positive (347)	8.6% (30)	91.4% (317)

### 2.2 Comparison to OCT Stalling

**Fig. 3:**
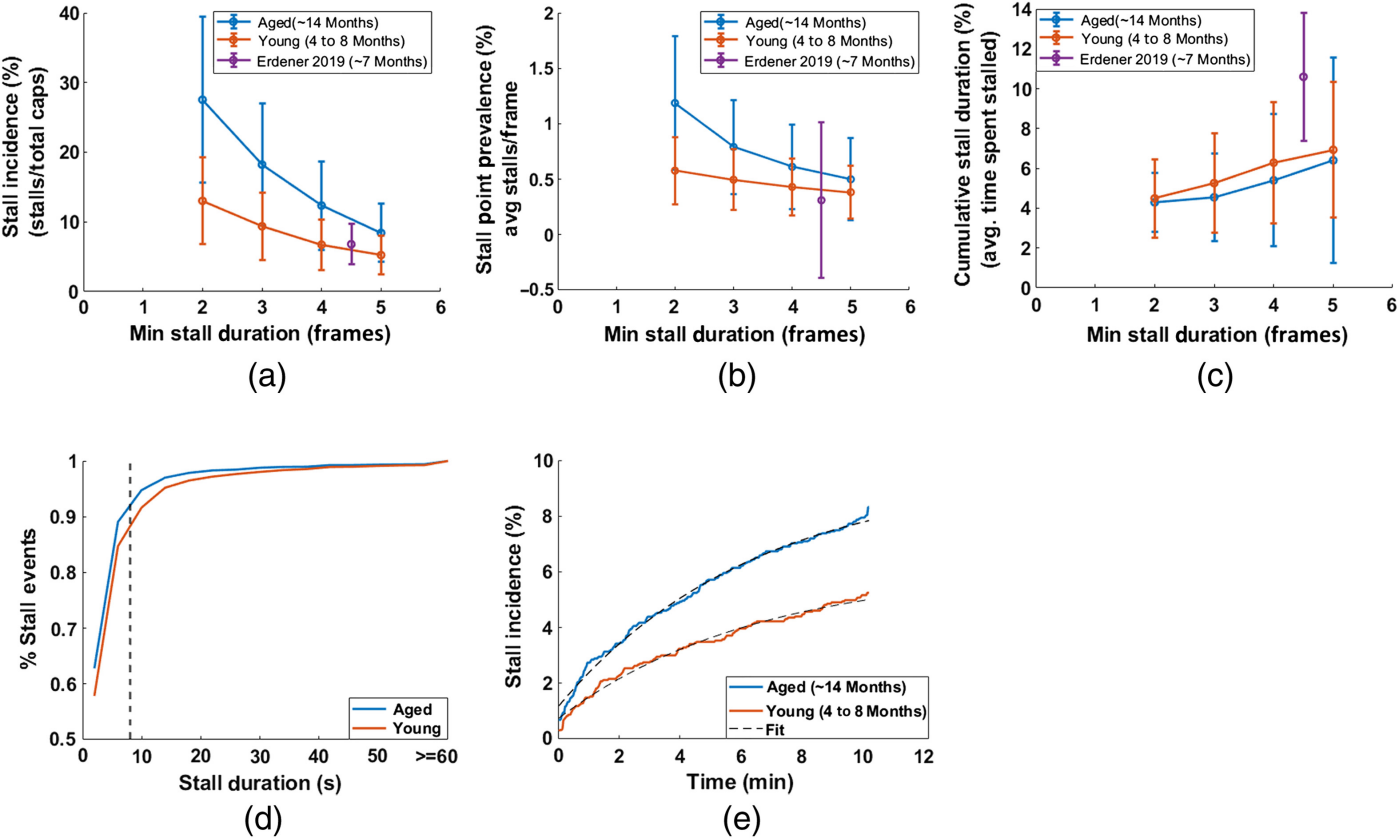
(a)–(c) Stalling statistics calculated after removing stalls shorter than a given length. Stall length of two includes all stalls detected. Purple point indicates the same statistics reported previously by OCT for a chronic window^13^ used to compare (a) stall incidence, (b) point prevalence, and (c) cumulative stall duration. (d) Cumulative distribution function of all individual stalling events of young and aged groups. Dashed line represents the time to generate a single OCT angiogram. (e) Stall incidence as a function of measurement time. Dashed black lines shows fit of the exponential model $I_{\text{stall}}=A(1-\exp(-\frac{B}{t}))+C)$. The best fit resulted in A=8.4, B=6.4, and C=1.15 for the aged group and A=5.4, B=6.5, and C=0.7 for the young group.

After validation of our stall analysis, we then compared our resulting stall parameters using the correlation aided detection to stalling statistics previously reported with OCT (Table 2).^13^ We found stalling incidence was twice that reported by OCT, likely due to the increased temporal resolution of the Bessel beam two-photon microscope. When looking at the distribution of the duration of the stalling events [Fig. 3(d)], most of the individual stalling events lasted <8  s and therefore would easily be missed OCT-based angiography, which takes ∼8  s to generate an image. This also contributes to the lower cumulative stall duration we measured with the Bessel beam microscope, as OCT was only sensitive to longer stalls and thus does not identify many capillaries that have only short duration stalling events [as suggested by Fig. 3(a)]. To confirm this, we removed short duration stalling events in our Bessel beam results and then recalculated the statistics. When we only consider stalls occurring for ∼8  s or longer, our measured incidence is very similar to what was found previously with OCT [Fig. 3(a), Table 2]. The cumulative stall duration [Fig. 3(c)] did remain lower, still likely due to the increased temporal resolution and improved ability to identify the end of longer stalling events with the Bessel beam approach. The variability in stall point prevalence found in OCT [Fig. 3(b)] was too high to make a precise comparison to that found with the Bessel beam results, but the results are none-the-less consistent.

**Table 2:** Comparison of stalling statistics measured by OCT (reported in Ref. 13) and by Bessel beam two-photon microscopy (BB-TPM) in two different age groups (4 to 8 months and 14 months). The long stalls only column calculates stall statistics for stalls lasting 5 Bessel frames or more (i.e., ∼8  s) to facilitate comparison with the previously published OCT results.

	OCT^13^ (∼7 months)	BB-TPM (4 to 8 months)	Long stalls only	BB-TPM (14 months)	Long stalls only
ROIs	12	10	10	14	14
Animals	6	4	4	4	4
Minimum stall duration (s)	∼8	1.75	∼8	1.75	∼8
Incidence (%)	6.8 ± 2.9	13.02± 6.2	5.24 ± 2.74	27.5 ± 12	8.4 ± 4.16
Point prevalence (%)	0.31 ± 0.7	0.58 ± 0.3	0.38 ± 0.23	1.18 ± 0.6	0.49 ± 0.37
Cumulative stall duration	10.6 ± 3.2	4.49 ± 1.97	6.91 ± 3.39	4.29 ± 1.49	6.4 ± 5.17

We then applied our correlation aided approach to analyze data taken from older (n=4 mice, 14-month-old) mice and compared it to the results from the younger group analyzed in the validation study. The older cohort had increased rates of stalling compared to the younger group, predominantly of shorter duration [Fig. 3(d)]. When only looking at longer duration stalls (more than 4 frames, i.e., close to ∼8  s), stall incidence was closer to the young cohort and previous OCT results, though still elevated. In both cases, the incidence of these long stalling capillaries over measurement time can be fit to a simple exponential model $I_{\text{stall}}=A(1-\exp(-\frac{B}{t}))+C)$, indicating a subset of capillaries consistently stall rather than being randomly distributed over the entire capillary network.

### 2.3 Photothrombotic Stroke

**Fig. 4:**
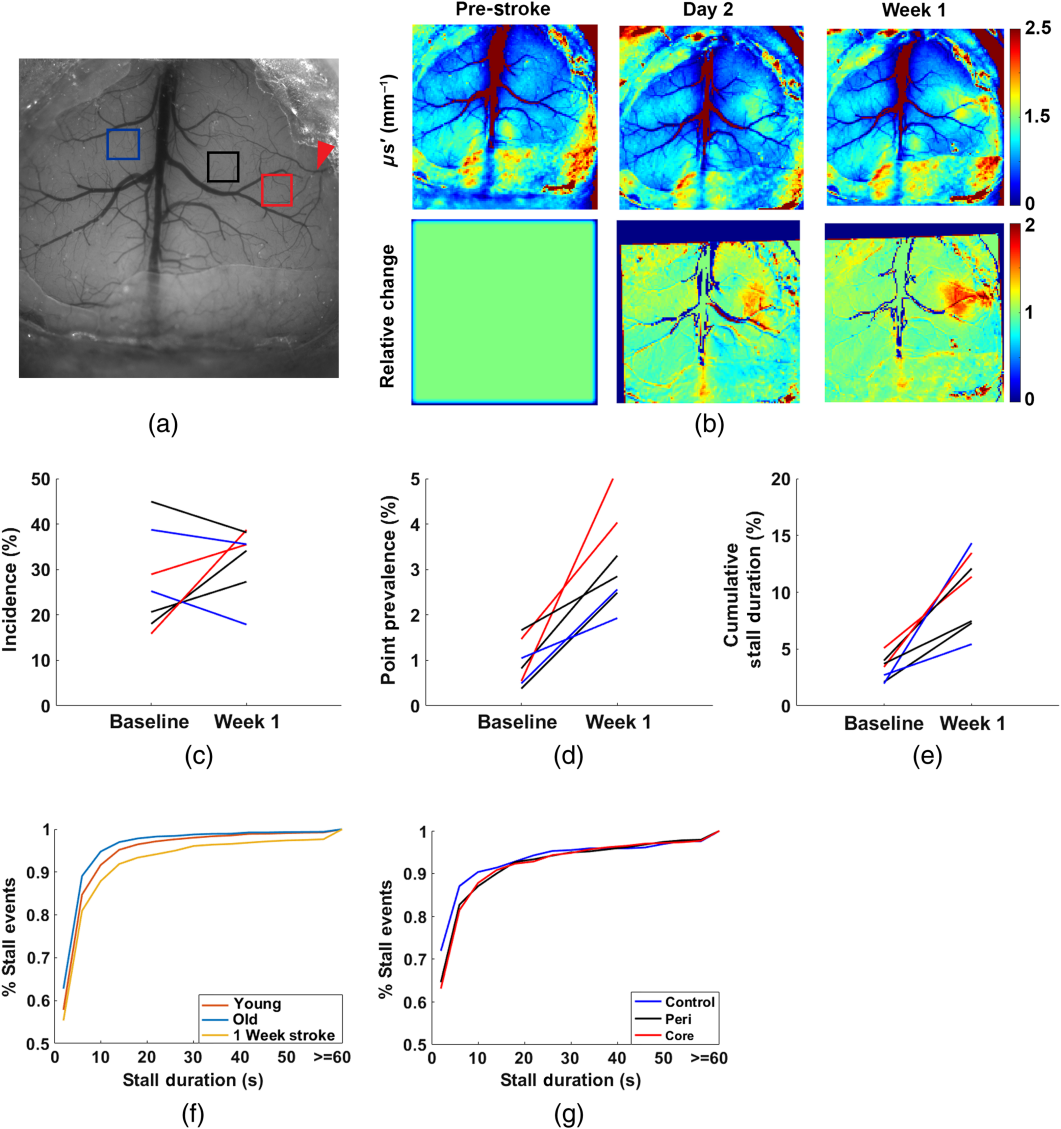
(a) Widefield reflectance image showing cortical vasculature at baseline. Red arrow indicates the artery that was targeted for photothrombotic stroke. Boxes show the ROIs that were imaged at baseline and 1 week post-stroke. Colors indicate the relation of the ROI to the stroke. Red indicates stroke core, black peri-infarct (peri), and blue contralesional hemisphere (control). (b) SFDI reduced scattering coefficient ($\mu_{s}^{\prime }$) and relative images show the increase in scattering as a result of the stroke, used to determine the stroke core. (c)–(e) Changes in stall statistics in 7 ROIs across 2 mice from baseline to 1 week after stroke. Color indicates the relation to the stroke core. (f) Cumulative percentage of stalling events across all stalling capillaries as a function of duration across different groups. Line indicates the percentage of stalls with a duration less than or equal to the duration. (g) Cumulative percentage of stalling events across stalling capillaries based on region at 1-week post-stroke (Video 2, mp4, 11.1 MB [URL: https://doi.org/10.1117/1.NPh.10.3.035009.s2]).

To demonstrate the ability of our system to track changes in stalling statistics, we used a targeted photothrombotic model of stroke^26^ and measured the differences in capillary stalling at baseline compared with 1 week post-stroke in 14-month-old mice. Regions-of-interest (ROIs) were determined to be in the stroke core, peri-infarct, and contralesional hemisphere as identified using spatial frequency domain imaging (SFDI) to track changes in tissue scattering [Fig. 4(b)].^27^ In total, there were three ROIs in the stroke core, three in the peri-infarct region, and three in the contralesional hemisphere across two animals. Both ROIs in the stroke core and all but one ROI in the peri-infarct region saw increased stalling incidence. The only ROI that did not show an increased incidence at week 1 also had the highest incidence at baseline. We also found an increased presence of longer stall durations in all ROIs, including the ROIs in the contralesional hemisphere [Figs. 4(e) and 4(f)]. The stalling point prevalence was also increased [Fig. 4(d)], primarily driven by stalling duration, as it increased even in regions where overall incidence was unchanged. This shift is also apparent when looking at the distribution of the durations of the stalling events across all capillaries [Fig. 4(f)]. At baseline, the older mice had higher incidence, which was primarily driven by shorter stalling events. One week after stroke, longer duration events account for a larger percent of all stalls, even in the contralesional control ROI [Fig. 4(g)].

### 2.4 Arterial Dilations Transiently Reduce Stalls

**Fig. 5:**
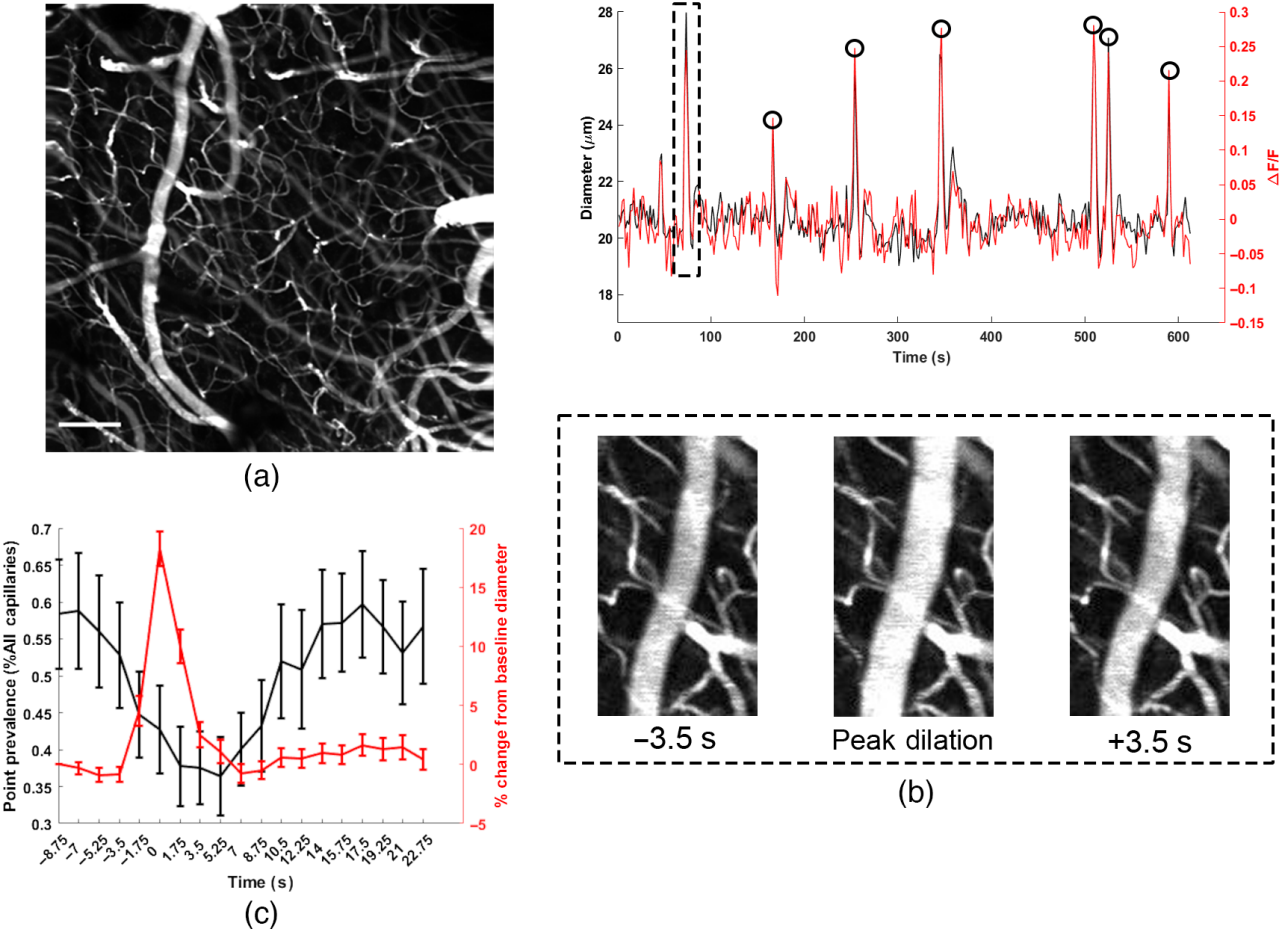
(a) Full stalling field of view with an artery on the left-hand side. Scale bar is 100  μm. (b) Diameter and fluorescence intensity (ΔF/F) trace of a section of the artery shows close correlation between calcium fluorescence and diameter with the Bessel beam (correlation coefficient: 0.88). Black circles indicate time points used for analysis in (c). (c) Changes in stall point prevalence (black) relative to large dilation events (red), averaged across 9 ROIs. Time 0 corresponds to the time of peak diameter during dilation. Error bars indicate the standard errors estimated across n=65 dilations from 9 ROIs.

In addition to volumetric imaging offered by the extended depth of field, Bessel beam allows for simple estimation of vessel diameter using the vessel fluorescent intensity^23^ [Fig. 5(b)]. We hypothesized that periods of increased flow may reduce stalls by providing sufficient pressure across the capillary to clear the stall. We therefore analyzed the average stalling point prevalence around large, isolated dilation events (see Sec. 4). We found that the average point prevalence was significantly reduced by the time of peak dilation [Fig. 5(c)] and shortly after. This decline started prior to the peak diameter because flow starts to increase as soon as vessel diameter starts to increase.

## 3 Discussion

To overcome the limited volume rates of conventional OCTA and two-photon angiography, we used a custom-built Bessel beam two-photon microscope to monitor a 713×713×120  μm volume at 0.57 Hz for instances of stalled capillary flow. This was 3 to 4 times faster than OCT angiography used in the previous studies to monitor a similar volume.^13^^,^^18^^,^^28^^,^^29^ The increased speed led to a bottleneck in analysis, which was previously done fully manually. We semi-automatically extracted intensity along the centerline of all capillaries of interest and used it to flag time points of interest in all capillaries. The high false positive rate meant that our correlation metric was not sufficiently robust to directly detect stalls, but it was enough to reduce the time burden of analysis by about half (from 12 to ∼6  h). It also identified the vast majority of stalling events, including many that blind observation missed. The latter is especially important as the results of the fully manual analysis indicated that observers missed as much as 70% of all the stalling events (Table 1), and that this manual analysis was adapted from the approach used to analyze OCT angiograms.^13^^,^^18^ This is understandable given the complexity and size of even a single dataset and given that it is easier to confirm that the automatically identified stalling events are truly a stalling event as opposed to the fully manual approach, which requires blind inspection of all capillaries at all time points. A similar metric could also be adapted to assist with the analysis of OCT angiograms.

While we were able to greatly reduce the analysis burden while improving accuracy, stalling analysis was still time-consuming. Deep-learning-based tools have shown promise in distinguishing these events from normal flowing capillaries,^30^ which could further reduce or eliminate the need for human observers. Due to the increased rate of stall detection with our approach compared with traditional two-photon microscopy, the relatively small number of datasets used in our studies still contain processed results from over 6000 capillaries that were monitored for 10 min each. Therefore, a sufficiently large training set of stalled and flowing capillaries could be created with fewer datasets compared to previous acquisition methods.^30^ Our analysis would also likely benefit from automated extraction of capillary traces from the Bessel beam data.^30^^–^^32^

We also leveraged the ability of Bessel beam to monitor pial artery dynamics simultaneously with capillary stalling [Fig. 5(a)]. As has been shown by others, vessel diameter correlates highly with vessel intensity^23^ [Fig. 5(b)], as a larger volume of intravascular dye is excited when the vessel diameter is increased. This provides an easy to calculate metric for vessel diameter that is insensitive to axial motion, which we used to observe drops in stalling point prevalence around large dilation events [Fig. 5(c)], as stalls were presumably cleared due to the transient increase in flow and perfusion pressure. This is consistent with recent work showing that environmental enrichment reduced the number of capillaries plugged by injected microspheres.^33^ But here we show that there is on average fewer stalled capillaries around larger arterial dilation events *in vivo*, demonstrating the potential for dynamic reductions in stalling from spontaneous flow changes, not just under direct functional activation.^13^ This effect was shown with large and isolated dilation events, but more sophisticated analysis can be done to better understand the relationship between stalling and neurovascular coupling, or even stalling and resting state oscillations.^34^

To demonstrate the ability of our approach to detect changes in stall characteristics, we compared the statistics between young and old mice to previously reported statistics.^13^ Similar to the previous work, we found that a subset of capillaries repeatedly stalled [Fig. 1(d) and Table 2], rather than being randomly distributed across the capillary network.^13^ The similarity in long stalling parameters [Table 2 and Fig. 3(e)] to OCTA gives confidence that we are observing the same events with the addition of shorter events OCTA could not capture previously. At baseline, the older mice had more than twice the number of capillaries that stalled at any given time (point prevalence) and in total (incidence) but had a larger share of stalling events that were shorter in duration [Fig. 4(f)] resulting in a lower cumulative stall duration. This is further confirmed when removing shorter stalling events from analysis, resulting in a stall incidence more similar to the younger group. The stall incidence of longer stalling events was consistent with that reported with OCT,^13^ where slower frame rates were used and thus would not be sensitive to shorter events. In the older group, two mice also underwent a photothrombotic model of stroke and were reimaged 1-week post-stroke. Stroke resulted in a significant rise in stalling point prevalence and cumulative stall duration. This indicates that there is an increase in the duration of stalling events, even in the contralesional hemisphere. When recomputing the stall statistics using only longer events, an increase in stall incidence is also observed (Fig. S1 in the Supplementary Material). Circulation of neutrophils can still be elevated at this 1 week stage in recovery,^35^ which could drive increases in stalling across the cerebral vascular network, including in the contralesional hemisphere. However, a larger scale study would be needed to confirm these phenomena. Future work can also address the source of longer versus shorter duration stalls using fluorescent labeling methods. For example, neutrophils adhering to the vessel wall could be the main driver of longer stalls as well as explain why only a subset of capillaries repeatedly stalls. This would be consistent with the previous work, where administering Anti-Ly6G to interfere with the adhesion of neutrophils reduced stalling.^14^^,^^18^ The question of whether neutrophils primarily drive longer stalls could be addressed with multicolor imaging using Rhodamine 6G as a second fluorophore^12^^,^^14^ (Video 2).

Our ability to detect shorter stalls also raises the question of the shortest stall duration that should be considered during analysis. Previous work was only sensitive to longer stalls due to the nature of the measurement approaches and showed that the reduction in long stalls was critical and increased total cerebral blood flow well in agreement with a computational model.^14^ In particular, it was found that a decrease in stalls from 1.8% to 0.72% of all capillaries resulted in a 13% increase in total cerebral blood flow.^14^ Similar modeling and analysis have not yet been done to account for brief stalls in flow, but modeling of capillary transit times has shown that overall flow heterogeneity in the capillary network reduced oxygen delivery efficiency, even for the same total cerebral blood flow.^19^^,^^36^ Increased stalling, even for brief periods, would lead to increased intracapillary flow heterogeneity and therefore be potentially detrimental despite their short duration. There is also predicted upstream and downstream effects of flow stoppages such that even brief stalls could theoretically increase spatial flow heterogeneity,^37^ meaning flow heterogeneity is created across the local network. The increase in short stalls we found in our aged mice would be consistent with the increased spatial capillary speed heterogeneity found in older mice, where pockets of hypoxic tissue were also found more frequently.^38^ On the other hand, while brief stalls are likely driving increases in flow heterogeneity that could lead to pockets of hypoxic tissue, the diffusion of oxygen from the capillary tissue acts as a low-pass filter of capillary oxygen dynamics^39^ and smooths out brief drops in oxygen, such that brief stalls might not induce critical levels of hypoxia in the adjacent tissue. Therefore, more work needs to be done to determine the impact of varying stall durations on oxygen delivery.

Since the imaging volume rate is equivalent to the imaging frame rate with Bessel beam two-photon microscopy, it offers the potential for imaging capillaries at very high-volume rates.^23^ The frame rate used in our study was relatively slow compared to what is achievable with even standard linear galvanometers but was used to allow for large field of view images with a high signal–to-noise ratio, ensuring a large number of capillaries can be monitored. It was also important to keep frame periods sufficiently long such that the presence of the same RBCs between frames [Fig. 1(b)] was indicative of stalled flow, as even small shifts in RBC position would still mean a flow speed on the order of μm/s instead of mm/s as is typically observed.^11^^,^^40^^,^^41^ Using higher frames rates (such as 100 to 1000 Hz^23^^,^^42^^,^^43^) where RBC flow can be tracked would require a speed cutoff for stall classification. Further work needs to be done to understand what duration of stall is required to significantly affect oxygen delivery, which would then inform the optimal temporal resolution or speed threshold for quantifying their dynamics. Regardless, future investigations into capillary stalling will greatly benefit from high speed, high throughput measurements of capillary stalling statistics. Bessel beam two-photon microscopy offers an ideal platform with an improved volume rate over conventional approaches and multicolor imaging of fluorescently labeled cells and plasma that can easily monitor hundreds of capillaries to study these events.

## 4 Methods

### 4.1 Animal Preparation

All procedures were reviewed and approved by Boston University Institutional Care and Use Committee. Animals were implanted with a custom titanium headpost and either a full crystal or two-half crystal skulls (LabMaker), as described previously.^44^^,^^45^ Animals were allowed to recover from surgery for 3 weeks before undergoing acclimation and imaging. During acclimation to head fixation, animals were head fixed for increasingly longer durations and given sweetened condensed milk as a treat. Animals were monitored for signs of distress and removed early as necessary to avoid excessive stress. Acclimation sessions started at 15 min and continued daily until animals were comfortable with head fixation for 90 min.

### 4.2 Two-Photon Microscopy

All imaging was performed under a home-built Bessel beam two-photon microscope with a 100  μm axial PSF. An axicon based Bessel module was used to generate an annular illumination focused at the back pupil plane of the objective (Olympus 25×, 1.0 NA). Microscope control and data acquisition were done using ScanImage^46^ (Vidrio Technologies). Fluorescence was excited at 920 nm with a tunable Ti:Sapphire laser (Insight X3, Spectra Physics) with an electro-optic modulator (Conoptics 305-105-20) for fast power control. The excitation and emission paths were split by a primary dichroic (Semrock DI03-R785), and fluorescence was collected and detected by two cooled photomultiplier tubes (PMTs) (Hamamatsu H74422-40 and -50). The fluorescence was split by a secondary dichroic (Semrock FF555-DI03) and passed through either a red (Semrock FF01-607/70) or green (Semrock FF01-502/60) before being detected by the PMTs.

### 4.3 In Vivo Imaging

Animals were briefly anesthetized and retro-orbitally injected with 30 to 50  μL of 150 kDa FITC-Dextran or Texas Red-Dextran (5% in PBS) and then head-fixed in a custom rig. The rig was then mounted on a motorized stage setup under the microscope. The custom rig used a goniometer stage for control of tip and tilt control on the headbar and craniotomy. The stage was adjusted such that the desired region of interest was perpendicular to the imaging plane of the microscope. Survey raster scans were used to check field flatness and match region of interests across sessions before acquisition of full times series. 512×512  pixel images of a 713×713×120  μm volume of view was scanned at 0.57 Hz for ∼10  min (350 total images) and saved for offline processing. During the imaging sessions, the mouse was checked every ∼30  min for signs of distress and given a treat of sweetened condense milk like in training sessions.

### 4.4 Data Processing

Time series data were saved as tif series directly from ScanImage and used for analysis. For manual processing, data were loaded into a custom MATLAB GUI similar to that used for OCT angiogram stall analysis.^13^^,^^18^ Using a zoom feature, a sub-ROI was focused on for analysis. The series was then inspected frame by frame looking for instances of stalling. Stalls were classified as frames where all RBC shadows that appear in the capillary were stationary between frames. Any movement in the RBCs or change in the number of visible RBCs was classified as flowing (non-stalled). Analysis was completed for each sub-ROI until all capillaries were observed. Once all stalled capillaries had been found, each stall was then analyzed frame by frame and all frames where the capillary stalled were manually annotated. The manually annotation as well as segmented capillary and centerline (see below) were then saved for group analysis. Finally, the total number of capillaries in the ROI was counted and recorded. Only capillaries with clear RBC contrast were counted.

For correlation-based detection, capillary centerlines were semi-automatically extracted by first marking all capillaries of interest with a single click on each capillary. Capillaries were selected based on having sufficient contrast to see RBC shadows along the length of the vessel. To extract centerlines, images first underwent vessel enhancement to increase the contrast of the thinner capillaries using the Frangi vesselness filter function fibermetric in MATLAB. Images were then thresholded for a black and white segmentation of vessels versus background and then a centerline skeleton was extracted using the bwskel function. The skeleton was converted to a graph following the approach used by Ji et al.^25^ The nearest edge in the graph to each manual click was then used at the capillary centerline. The intensity along the centerline was then extracted frame by frame and then used to generate the LT images similar to a kymograph. To reduce the effects of motion on the LT images, an image was cropped around each centerline and then locally registered across time. This transform was then applied to the centerline to keep it centered on the vessel even during and after motion.

### 4.5 Vessel Diameter

To estimate vessel diameter, 20 lines spaced 2  μm apart were drawn along the width of the vessel. The resulting intensity profiles were then averaged and normalized. The diameter was estimated to be the full-width at half-max of the resulting profile. Fluorescent intensity was measured using a small rectangular ROI drawn inside the same vessel, and mean intensity was used to calculate ΔF/F.

Large dilation events were identified manually from 9 of the 10 ROIs from the validation dataset after extraction of vessel dynamics. One ROI was excluded because it lacked any significant vasodilation events. To prevent the potential confound of successive dilation events, we only analyzed isolated dilation events. Events where another large vasodilation occurred within 10 frames (17.5 s) were excluded.

### 4.6 Validation Experiment

Validation was performed on 10 datasets taken in healthy 4- to 8-month-old mice (C/57BL/6J; Jackson Labs) that had already undergone fully manual analysis. Capillaries were selected as described above and LT plots generated. Capillary centerlines of 5 pixels or less were excluded from analysis. For each time point (row) in the image, the intensity was correlated with the intensity of the next frame (row), resulting in the frame-to-frame vessel intensity correlation over time. This correlation trace was thresholded, and all suprathreshold time points were flagged as stalls. A new observer (different from the observer who conducted fully manual analysis) then inspected every flagged result and marked it as a true or false positive. The new observer also inspected stalls marked during fully manual analysis, including those not flagged by the correlation metric. The analysis was restricted to only flagged capillaries and time points to minimize the time burden of analysis.

### 4.7 Photothrombotic Stroke

Stroke was induced following a vessel-targeted photothrombotic model^26^ to provide a clinically relevant model. The stroke was performed under a custom built intrinsic optical signal imaging and laser speckle contrast imaging system that allowed for continuous flow monitoring during occlusion.

The location of the stroke was determined using SFDI^27^ taken on day 2 and on day 7 in a short session prior to Bessel imaging. ROIs at baseline were selected based on the region the target vessel fed and were predicted to be close to the stroke boundary. One ROI was also taken in the contralateral hemisphere to serve as a control. Each ROI was then reimaged 1 week after the stroke for comparison to baseline and classified based on proximity to the stroke core. Strokes were performed on four 14-month-old C57BL/6J-Tg(Thy1-GCaMP6f)GP5.17Dkim//J (Jackson Labs). Two of the animals did not have successful occlusion during the photothrombotic procedure and were excluded from the 1-week follow-up. Baseline results were pooled and used as comparison younger cohort and OCT.

## Data Availability

Analysis code is available in Boston University Neurophotonics GitHub. Datasets can be found at DOI: 10.6084/m9.figshare.23902137.
