# Supplementary material for: High throughput detection of capillary stalling events with Bessel beam two-photon microscopy
Source: Neurophotonics. 2023 Sep 12;10(3):035009. doi: 10.1117/1.NPh.10.3.035009 (PMC10495839; doi:10.1117/1.NPh.10.3.035009)
Supplement: Supplementary file 1 [file NPh_010_035009_SD001.pdf]

Supplemental Material

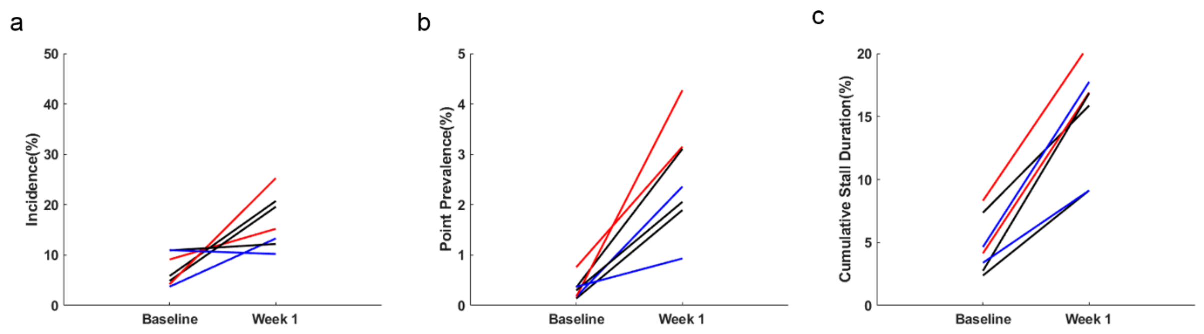

Supplemental Figure 1 Stall statistics at baseline and one week post stroke calculated using only longer duration stalling events. Colors indicate region relative to the stroke (red indicates stroke core, black peri-infarct, and blue contralesional hemisphere) (a) Stall incidence (b) Stall point prevalence (c) Cumulative stall duration

Supplemental Movie 1 Representative time series of data used to capture stalling events

Supplemental Movie 2 Time series of a Rhodamine-6G labeled leukocyte (yellow) plugging a capillary (red) bifurcation
